# Sleep disorder or simple sleep ontogeny? Tendency for morningness is associated with worse sleep quality in the elderly

**DOI:** 10.1590/1414-431X20165311

**Published:** 2016-10-10

**Authors:** A.A. Barbosa, M.A.L. Miguel, S. Tufik, F.C. Sabino, M.S. Cendoroglo, M. Pedrazzoli

**Affiliations:** 1 Departamento de Psicobiologia, Universidade Federal de São Paulo, São Paulo, SP, Brasil; 2 Departamento de Fisiologia, Universidade Federal do Rio Grande do Norte, Natal, RN, Brasil; 3 Departamento de Geriatria, Universidade Federal de São Paulo, São Paulo, SP, Brasil; 4 Escola de Artes, Ciências e Humanidades, Universidade de São Paulo, São Paulo, SP, Brasil

**Keywords:** Elderly, Sleep quality, Circadian rhythmicity, Chronotype, Sleep ontogeny

## Abstract

The objective of this study was to evaluate the alterations in sleep and circadian parameters during the aging process. The study sample comprises volunteers older than 18 up to 90 years of age that answered the Pittsburgh Sleep Quality Index (PSQI) and the Horne and Östberg circadian preference questionnaire. We observed that the shift to morningness with increasing age is associated with a significant worsening in sleep quality. We discuss that this sleep profile characterized by morningness and worse sleep quality observed in elderly, when compared to younger people, reflects not necessarily a pathological state, but an expected profile for this age group.

## Introduction

Individual differences in the sleep-wake behavior, encompassing aspects such as habitual bedtime, wake time, sleep duration and structure have been observed in several studies (1). Moreover, changes in sleep parameters have been associated with aging even in healthy people without sleep disorders (2).

It has been proposed that sleep is regulated by two processes: circadian and homeostatic. These two processes are generated independently, but their interaction regulates sleep-wake cycles and from it emerges the sleep phenotypes and sleep-wake disorders (3). Easily observable phenotypes that are associated with these processes include chronotype or diurnal preference and sleep fragmentation. Many different instruments have been developed to measure sleep quality and diurnal preference. Sleep quality can be defined subjectively by self-reporting or by more objective measures, such as polysomnography or actigraphy (4). Questionnaires are convenient and efficient instruments for evaluating sleep-rating measures and diurnal preference. One of the most widely used questionnaires is the Pittsburgh Sleep Quality Index (PSQI) (4), a self-rated questionnaire with nineteen questions, which assesses seven components of sleep quality and disturbances from the month prior to completing the questionnaire. The questions evaluate subjective sleep quality, sleep latency, sleep duration, habitual sleep efficiency, sleep disturbances, use of sleeping medication, and daytime dysfunction (5).

Self-reported diurnal preference can be measured by the Horne and Östberg (HO) questionnaire (6), which has been used extensively in several studies for the last 30 years. The circadian types are categorized as morning-types or "larks", evening-types or "owls", and types in-between these or intermediate-types. Morning-types wake up early and go to sleep early, while evening-types are active during the early night and cannot wake up early easily. This morningness-eveningness phenotype represents the extremes in diurnal preference (6).

Sleep patterns change ontogenetically and are very different in older people. Reports of oral temperature measurements have shown evidence of a weakened 24-h periodicity in the elderly, even in those individuals with generally regular lifestyles (7). Compared to younger subjects, older subjects present sleep consolidation differences characterized by earlier habitual bedtime and wake time, phase advance of the body temperature rhythm with a tendency for the minimum circadian temperature rhythm to occur earlier in the night and a more disturbed sleep (8). The decline in sleep quality that often accompanies aging is thought to be a consequence of alterations in both circadian and homeostatic processes, which are widely assumed to be responsible for sleep/wake regulation (9).

In spite of its biological and medical relevance, few studies have been specifically designed to correlate sleep quality and diurnal preference in an ontogenetic perspective. Thus, the aim of the present study was to evaluate concomitantly the alterations in sleep and circadian parameters during the aging process. Specifically, this report evaluated the association between changes in sleep quality and chronotype throughout the aging process.

## Material and Methods

### Participants

We contacted the volunteers in schools, universities, and companies, such as clinical laboratories and offices, and explained the purpose of this study. After a verbal explanation, their written consent was obtained. One thousand and thirty-five volunteers living in São Paulo, SP, Brazil, were initially recruited to answer to the PSQI and HO questionnaires.

### Selection

Participants older than 18 years of age, of both genders and who had answered all the questions in both questionnaires, were drawn from the initial recruitment, resulting in a final sample of 812 volunteers. All participants belonged to the Brazilian population, which is very ethnically interbred, and consists mainly of European (Portuguese) and Brazilian aboriginal backgrounds that were later mixed with a variety of African groups and with a variety of European ethnicities (mainly Italian and Spanish) and Asiatic ethnicities (mainly Japanese) at the beginning of the 20th century (10).

The study was approved by the Ethics Committee of Universidade Federal de São Paulo and informed consent was obtained from the participants (#CEP 1471/07).

### PSQI

The PSQI is a self-rated questionnaire that assesses the sleep quality and disturbances of an individual over the month prior to completing the questionnaire. The nineteen questions are combined into seven clinically derived component scores, each weighed equally from 0–3. The sum of scores for these seven components yields one global score ranging from 0–21, with higher scores indicating worse sleep quality (4). To assess sleep, we used the Brazilian version of the PSQI (5).

### Diurnal preference

The Horne-Östberg (HO-MEQ) questionnaire provides a quantitative measure of diurnal preference (6), which has been validated to correlate with the timing of the core body temperature and melatonin rhythms (11). In this study, the data were collected through a Portuguese version of the HO-MEQ (12).

### Age categorization

To analyze answers from HO and PSQI questionnaires, the volunteers were classified according to their self-reported age in 10-year categories, except for the flanks of the distribution. They were categorized as the following age ranges, respecting adolescence and elderly age concepts: 18 to 24 (group 1), 25 to 34 (group 2), 35 to 44 (group 3), 45 to 54 (group 4), 55 to 64 (group 5), and 65 years old and older (group 6).

### Statistical analyses

We used ANOVA and ANCOVA to analyze differences among age groups with HO scores as a covariate. *Post hoc* analyses were performed using the Fisher test. Spearman's test was used to analyze the correlations between PSQI index and HO score, and the correlations between each of them with age. Multiple correlation was used to analyze the PSQI index with age and HO as predictors. The level of significance was set at P<0.05.

## Results

The sample was composed by 812 individuals, average age of 39.7 years (18–94 years), and 64.6% were women. The male/female proportion, the number of subjects, the mean values for HO scores and PSQI by age group, are shown in Table 1.

**Table:**
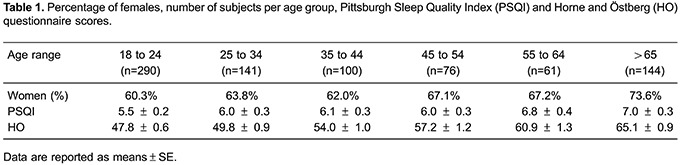

Chronotype and sleep quality correlated with age (r=0.50 and r=0.13 respectively, P<0.05), however multiple correlation considering age and HO scores as predictors of sleep quality reveals a stronger correlation (r=0.28 and P<10^-6^). In general, considering the HO score distribution by age, late chronotypes (evening types) presented worse sleep quality (r= -0.11 and P<0.05).

**Figure 1:**
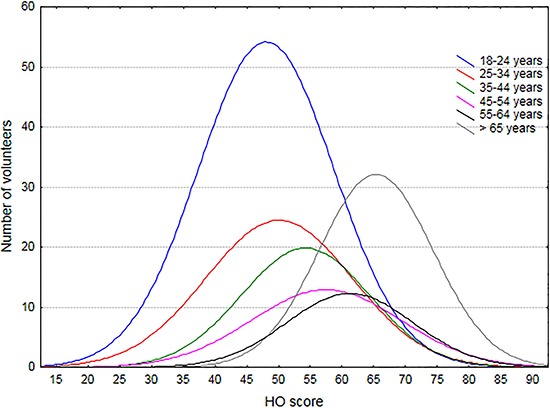
Horne and Östberg (HO) questionnaire score distribution according to age group. Higher scores indicate greater morningness.

When we analyzed the distribution of HO scores according to the age categories, we observed a clear and progressive shift to morningness with increasing age (ANOVA F(5, 806)=63,156, P<0.001). Fisher *post hoc* test confirmed significant differences between groups, except when group 1 (18 to 24 years old) and group 2 (25 to 34 years old) were compared. Figure 1 shows HO score distributions according to age groups.

**Figure 2:**
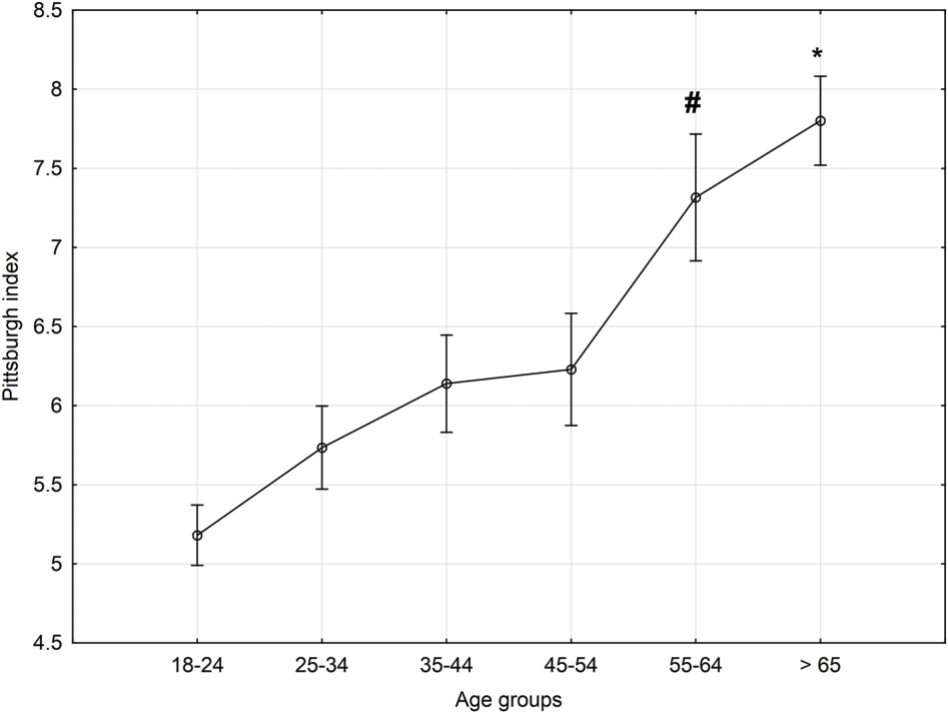
Averaged Pittsburgh Sleep Quality Index per age group. Data are reported as means±SE for each age range. Covariate means (HO score): 53.89. *P<0.05 compared to the other groups, except the 55–64 group; #P<0.05 compared to the 18–24 group (Fisher LSD test).

Additionally, considering HO score as a covariant, we observed that after 65 years of age (group 6), there was a significant worsening in sleep quality (F(5, 805)=11,864, (P=10^-6^, HO covariate mean: 53.9, Figure 2).

## Discussion

The analyses of the present results reveal that, ontogenetically, there is a strong tendency for morningness and worsening sleep quality with increasing age. Interestingly, the chronotype progressively changed with increasing age, while the worsening of subjective sleep quality started to change more abruptly beyond 55, and became worse after 65 years of age.

Ontogenetic changes in chronotype have been widely reported. In general, it is plausible to state that younger individuals have a strong tendency toward eveningness, whereas aging is strongly associated with morningness (13–15). In healthy elderly individuals without sleep disorders, the parameters of the circadian rhythms of sleep-wake (sleep onset and offset), melatonin (onset), core body temperature (acrophase) and cortisol (acrophase) occur earlier in the day, when compared to young adults (16,17). Other changes in this age group include reduction in the amplitude of circadian rhythms and reduced tolerance to abrupt phase changes (18). It is suggested that the chronotype remains relatively stable until around the age of 35 and morningness increases afterwards (19,20). In addition to changes in chronotype, sleep quality also changes as a function of ordinary aging process (21,22). Moreover, more than 50% of older adults (above 65 years old) have at least one chronic sleep complaint, the most prevalent being the inability to stay asleep at night (23). Thus, the sleep quality decline and the tendency towards morningness that were associated with increasing age in our sample are in agreement with previous reports (24–27).

Remarkably, few studies have been specifically designed to correlate sleep quality and diurnal preference (28). Based on studies focused on adolescents and young adults, eveningness has been associated with lower sleep quality, when compared with morningness (29,30). Daytime sleepiness, maladaptive sleep beliefs and irregular sleep-wake habits are also common traces attributed more frequently to evening-oriented people (31–33). Observing the overall data, the correlation between sleep quality and age is more evident when considering HO score as a predictor (controlling for HO score), which helps to uncover the worsening of sleep quality in the oldest age group.

These observations raise the question of whether morningness and worse sleep quality in the oldest individuals are independent phenomena or if they are related to each other. Classically, circadian and homeostatic controlling processes of sleep timing and duration have been described to be separately regulated (34). However, at first it was considered that these processes interacted at discrete moments. For example, a consolidated sleep episode is triggered when sleep debit is high and approaching upper circadian threshold. However, in recent years, a complex and constant reciprocal interaction between both systems in regulating sleep has been reported, which includes the sharing of neural networks, genetic components and regulation of neurotransmission (35,36).

Therefore, one can hypothesize that the age-sensitive, non-pathological neural/genetic/neurotransmission networks involved in the regulation of sleep and circadian rhythms interact and from this interaction emerges the sleep timing and duration profile normally observed in aged people. Typically, this profile is characterized by decreases in sleep time, less consolidated sleep, increases in the number and duration of nocturnal sleep awakenings (24) and significant changes in the amplitude of circadian rhythms (26). In older people, the sleep-wake cycle is not only entrained to an earlier clock time but also to an earlier endogenous circadian phase of the temperature cycle (37). Body core temperature rhythms and melatonin secretion are generally phase-advanced, and their intrinsic periodicity is often shortened (9,11).

Some studies have reported that PSQI is a useful and consistent tool for evaluating sleep quality in older adults (38). Epidemiological studies using PSQI have shown that a large number (65%) of individuals present difficulty falling or staying asleep. Additionally, less than 20% rarely or never reports complaints, demonstrating that the prevalence of sleep complaints is high, even in healthy older adults (23). These numbers are important to emphasize that sleep quality should be considered as a complex construct. To properly study this construct, distinct methods, with objective and subjective measures, have been developed (39). Despite emerging as a gold standard subjective tool for sleep quality estimation, PSQI does not correlate well with polysomnography, possibly because of the need for optimal cognitive function to answer the questionnaire (4). Furthermore, according to Landry et al. (40), more than 50% of the older adults (above 55 years old) who completed the PSQI can be classified as under-estimators of sleep quality, suggesting that they might tend to perceive sleep as being worse than it actually is. Thus, unless we consider almost every elderly individual as having a sleep disorder, it is unlikely that the PSQI is indicative of a real pathological state.

There were a couple of key limitations in this study. First, despite the difficulty to systematically collect objective measures of sleep in a large sample of volunteers, the adoption of an objective tool for the evaluation of other dimensions of sleep quality, such as actimetry, would provide means to test the prevalence of under-estimators of sleep quality in our senior's sample. Moreover, the prevalence of confounders in the elderly, such as sleep apnea, medication and psychiatric diseases, was not covered in this study. In addition, we should stress that, by no means, the associations described here between HO score and PSQI, indicates causality. Finally, this was a cross-sectional study and further longitudinal studies are needed in order to address changes in the association between circadian and homeostatic mechanisms of sleep-wake control.

In summary, with the subjective assessment of the ontogeny of chronotype and sleep quality combination, we observed that HO score was a good predictor of sleep quality. The chronotype distribution by age range showed that late circadian rhythms (represented by lower scores in each age range) are related to worse quality of sleep in all ages. Moreover, two extreme sleep profiles emerged as representative of the ontogeny of homeostatic and circadian sleep regulation; morningness and worse quality of sleep are characteristic of the elderly while eveningness and better quality of sleep are characteristic of young people.

Considering other reports in the literature (2,23), it is possible to argue or to propose that morningness and the sleep profile of aged people are emerging phenomena associated with a particular integration state of the neural/genetic networks regulating sleep and circadian rhythms at specific ages. Also, the characteristic sleep profile of older people does not necessarily indicate a pathological state, which can be a possible explanation for our results.

## References

[1] Barclay NL, Eley TC, Buysse DJ, Archer SN, Gregory AM (2010). Diurnal preference and sleep quality: same genes? A study of young
adult twins. Chronobiol Int.

[2] Vitiello MV, Larsen LH, Moe KE (2004). Age-related sleep change: Gender and estrogen effects on the
subjective-objective sleep quality relationships of healthy, noncomplaining older
men and women. J Psychosom Res.

[3] Franken P, Dijk DJ (2009). Circadian clock genes and sleep homeostasis. Eur J Neurosci.

[4] Buysse DJ, Reynolds CF, Monk TH, Berman SR, Kupfer DJ (1989). The Pittsburgh Sleep Quality Index: a new instrument for psychiatric
practice and research. Psychiatry Res.

[5] Bertolazi AN, Fagondes SC, Hoff LS, Dartora EG, Miozzo IC, de Barba ME (2011). Validation of the Brazilian Portuguese version of the Pittsburgh Sleep
Quality Index. Sleep Med.

[6] Horne JA, Östberg O (1976). A self-assessment questionnaire to determine morningness-eveningness
in human circadian rhythms. Int J Chronobiol.

[7] Kramer CJ, Kerkhof GA, Hofman WF (1999). Age differences in sleep-wake behavior under natural
conditions. Pers Individ Dif.

[8] Carrier J, Monk TH, Reynolds CF, Buysse DJ, Kupfer DJ (1999). Are age differences in sleep due to phase differences in the output of
the circadian timing system?. Chronobiol Int.

[9] Campbell SS, Murphy PJ (2007). The nature of spontaneous sleep across adulthood. J Sleep Res.

[10] Barbosa AA, Pedrazzoli M, Koike BD, Tufik S (2010). Do Caucasian and Asian clocks tick differently?. Braz J Med Biol Res.

[11] Duffy JF, Dijk DJ, Hall EF, Czeisler CA (1999). Relationship of endogenous circadian melatonin and temperature rhythms
to self-reported preference for morning or evening activity in young and older
people. J Investig Med.

[12] Benedito-Silva AA, Menna-Barreto L, Marques N, Tenreiro S (1990). A self-assessment questionnaire for the determination of
morningness-eveningness types in Brazil. Prog Clin Biol Res.

[13] Andrade MM, Benedito-Silva AA, Menna-Barreto L (1992). Correlations between morningness-eveningness character, sleep habits
and temperature rhythm in adolescents. Braz J Med Biol Res.

[B14] Roenneberg T, Kuehnle T, Pramstaller PP, Ricken J, Havel M, Guth A (2004). A marker for the end of adolescence. Curr Biol.

[15] Duarte LL, Menna-Barreto L, Miguel MA, Louzada F, Araujo J, Alam M (2014). Chronotype ontogeny related to gender. Braz J Med Biol Res.

[16] Carrier J, Monk TH, Buysse DJ, Kupfer DJ (1997). Sleep and morningness-eveningness in the 'middle' years of life (20–59
y). J Sleep Res.

[17] Duffy JF, Dijk DJ, Klerman EB, Czeisler CA (1998). Later endogenous circadian temperature nadir relative to an earlier
wake time in older people. Am J Physiol.

[18] Monk TH, Buysse DJ, Reynolds CF, Kupfer DJ, Houck PR (1995). Circadian temperature rhythms of older people. Exp Gerontol.

[19] Caci H, Deschaux O, Adan A, Natale V (2009). Comparing three morningness scales: age and gender effects, structure
and cut-off criteria. Sleep Med.

[20] Cofer LF, Grice JP, Sethre-Hofstad L, Radi CJ, Zimmermann LK, Palmer-Seal D (1999). Developmental perspectives on morningness-eveningness and social
interactions. Human Develop.

[21] Espiritu JR (2008). Aging-related sleep changes. Clin Geriatr Med.

[22] Crowley K (2011). Sleep and sleep disorders in older adults. Neuropsychol Rev.

[23] Foley DJ, Monjan AA, Brown SL, Simonsick EM, Wallace RB, Blazer DG (1995). Sleep complaints among elderly persons: an epidemiologic study of
three communities. Sleep.

[24] Buysse DJ, Reynolds CF, Monk TH, Hoch CC, Yeager AL, Kupfer DJ (1991). Quantification of subjective sleep quality in healthy elderly men and
women using the Pittsburgh Sleep Quality Index (PSQI). Sleep.

[B25] Jones KH, Ellis J, von Schantz M, Skene DJ, Dijk DJ, Archer SN (2007). Age-related change in the association between a polymorphism in the
PER3 gene and preferred timing of sleep and waking activities. J Sleep Res.

[B26] Taillard J, Philip P, Chastang JF, Bioulac B (2004). Validation of Horne and Östberg morningness-eveningness questionnaire
in a middle-aged population of French workers. J Biol Rhythms.

[27] Paine SJ, Gander PH, Travier N (2006). The epidemiology of morningness/eveningness: influence of age, gender,
ethnicity, and socioeconomic factors in adults (30–49 years). J Biol Rhythms.

[28] Chung MH, Chang FM, Yang CC, Kuo TB, Hsu N (2009). Sleep quality and morningness-eveningness of shift
nurses. J Clin Nurs.

[29] Selvi Y, Aydin A, Gulec M, Boysan M, Besiroglu L, Ozdemir PG (2012). Comparison of dream anxiety and subjective sleep quality between
chronotypes. Sleep Biol Rhythms.

[30] Roeser K, Meule A, Schwerdtle B, Kubler A, Schlarb AA (2012). Subjective sleep quality exclusively mediates the relationship between
morningness-eveningness preference and self-perceived stress
response. Chronobiol Int.

[31] Taillard J, Philip P, Coste O, Sagaspe P, Bioulac B (2003). The circadian and homeostatic modulation of sleep pressure during
wakefulness differs between morning and evening chronotypes. J Sleep Res.

[B32] Taillard J, Philip P, Bioulac B (1999). Morningness/eveningness and the need for sleep. J Sleep Res.

[33] Adan A, Fabbri M, Natale V, Prat G (2006). Sleep Beliefs Scale (SBS) and circadian typology. J Sleep Res.

[34] Borbely AA, Achermann P (1999). Sleep homeostasis and models of sleep regulation. J Biol Rhythms.

[35] Dijk DJ, Lockley SW (2002). Integration of human sleep-wake regulation and circadian
rhythmicity. J Appl Physiol.

[36] Borbely AA, Daan S, Wirz-Justice A, Deboer T (2016). The two-process model of sleep regulation: a
reappraisal. J Sleep Res.

[37] Dijk DJ, Duffy JF, Riel E, Shanahan TL, Czeisler CA (1999). Ageing and the circadian and homeostatic regulation of human sleep
during forced desynchrony of rest, melatonin and temperature
rhythms. J Physiol.

[38] Spira AP, Beaudreau SA, Stone KL, Kezirian EJ, Lui LY, Redline S (2012). Reliability and validity of the Pittsburgh Sleep Quality Index and the
Epworth Sleepiness Scale in older men. J Gerontol A Biol Sci Med Sci.

[39] Monk TH, Buysse DJ, Schlarb JE, Beach SR (2012). Timing, duration and quality of sleep, and level of daytime sleepiness
in 1166 retired seniors. Healthy Aging Clin Care Elder.

[40] Landry GJ, Best JR, Liu-Ambrose T (2015). Measuring sleep quality in older adults: a comparison using subjective
and objective methods. Front Aging Neurosci.

